# Targeting chemokines for acute lymphoblastic leukemia therapy

**DOI:** 10.1186/s13045-021-01060-y

**Published:** 2021-03-20

**Authors:** Zixi Hong, Zimeng Wei, Tian Xie, Lin Fu, Jiaxing Sun, Fuling Zhou, Muhammad Jamal, Qiuping Zhang, Liang Shao

**Affiliations:** 1 Department of Hematology, Zhongnan Hospital of Wuhan University, Wuhan, China; 2 Department of Immunology, School of Basic Medical Science, Wuhan University, Wuhan, China; 3 The First Clinical School of Wuhan University, Wuhan, China

**Keywords:** Acute lymphoblastic leukemia, Chemokine, Microenvironment, Therapeutic targets

## Abstract

Acute lymphoblastic leukemia (ALL) is a hematological malignancy characterized by the malignant clonal expansion of lymphoid hematopoietic precursors. It is regulated by various signaling molecules such as cytokines and adhesion molecules in its microenvironment. Chemokines are chemotactic cytokines that regulate migration, positioning and interactions of cells. Many chemokine axes such as CXCL12/CXCR4 and CCL25/CCR9 have been proved to play important roles in leukemia microenvironment and further affect ALL outcomes. In this review, we summarize the chemokines that are involved in ALL progression and elaborate on their roles and mechanisms in leukemia cell proliferation, infiltration, drug resistance and disease relapse. We also discuss the potential of targeting chemokine axes for ALL treatments, since many related inhibitors have shown promising efficacy in preclinical trials, and some of them have entered clinical trials.

## Background

Acute lymphoblastic leukemia (ALL) is the most common cancer among children with 25% chance of disease relapse [1], while in adults the chance is much higher [2]. According to the cell of origin, ALL can be further subclassified into B-ALL and T-ALL [3]. In clinical, B-ALL accounts for about 80% of pediatric leukemia where it is by far the most common malignancy, with a peak incidence around 2–5 years of age [4], but only accounts for 20% of adult leukemia. By contrast, T-ALL accounts for 10–15% and 25% in pediatric and adult ALL, respectively [5]. The five-year overall survival (OS) is about 85% and 40% in pediatric and adult B-ALL patients, respectively [6–9]. The 5-year event-free survival (EFS) of pediatric T-ALL is 70–75%, while it is 30–40% for adult T-ALL below 60 years of age, and only 10% above this age with intensified multi-agent chemotherapy [10]. Despite the progress in optimization of chemotherapy and the availability of hematopoietic stem cell transplantation, there is still a considerable scope for improving therapeutic outcome by developing novel biomarkers that can predict and refine prognosis in ALL patients.

Chemokines and their receptors have shown great potential in tumor-targeted therapy due to their active roles in remodeling tumor microenvironment [11]. Leukemia cells “hijack” the proliferative vascular niche and inhibit the osteoblastic niche to reshape the healthy bone marrow (BM) microenvironment into “leukemic” microenvironment, thereby destroying normal hematopoietic function and promoting its own proliferation and survival. Chemoresistance and relapse of acute leukemias are believed to be driven by leukemia initiating cells (LICs), which at least are partly dependent on signals from leukemic microenvironment to evade chemotherapy-induced death and acquire drug resistance [4]. With the progress of understanding the roles of signals like chemokines and their receptors in ALL, the use of chemokines or their receptors as novel targets to regulate leukemia cellular signal pathways will become a powerful new method for the therapy of ALL.

In this review, we aim to elaborate on the roles of chemokines and their receptors in ALL leukemic microenvironment, and how they contribute to the process of leukemogenesis and chemotherapy resistance. We also conclude potential chemokine-targeted ALL treatments and provide new insights into novel targeted therapy.

## ALL and its microenvironment

ALL is a malignant clonal disease associated with the abnormal proliferation of hematopoietic stem cells (HSCs) and progenitor cells. In ALL, abnormal progenitor cells and immature cells multiply rapidly in the BM, accumulate in the BM and inhibit normal hematopoietic function, and eventually they may infiltrate extramedullary organs such as the central nervous system (CNS), liver, spleen and lymph nodes.

It has been clearly confirmed that BM microenvironment plays a significant role in the occurrence and development of ALL, whose cellular composition includes HSCs, endothelial cells, osteoblasts, osteoclasts and mesenchymal stem cells (MSCs) [12]. BM microenvironment which includes osteoblastic (endosteal) and vascular niches secretes specific factors and then regulates the number and status of healthy HSCs [13–15]. The osteoblastic niches located in the endosteum are mainly composed of osteoblasts, osteoclasts and glial unmyelinated Schwann cells and regulatory T (Treg) cells. The vascular niches in the sinusoidal walls are mainly composed of CXCL12-abundant reticular cells, endothelial cells, nestin-positive MSCs and leptin receptor positive perivascular stromal cells. However, these niches also provide shelter where subsets of leukemic cells escape chemotherapy-induced death and acquire a drug-resistant phenotype, and leukemia cells are able to remodel the BM niches into malignant niches which better support neoplastic cell survival and proliferation [4]. Chemokines and their receptors play an important role in this process. For example, the stimulation of CXCR4 by CXCL12 produced by stromal cells in the BM is important for the maintenance of T-ALL cells [16].

In addition to the extensively studied BM microenvironment, the extramedullary microenvironments are also critical in the development and relapse of ALL [17]. CNS can protect blast cells from systemic chemotherapy [18], resulting in 10–30% of CNS relapse [19]. In T-ALL, chemokine receptors CCR7 and CXCR4 were associated with CNS infiltration of T-ALL cells [20, 21]. Furthermore, the hepatic microenvironment also provides an extramedullary niche for leukemic cells, which are maintained by the CXCL12/CXCR4 axis [22]. In splenic microenvironment, T-ALL cells could be stimulated to express a higher level of MIP-3β (CCL19), a ligand of CCR7, which further stimulates the proliferation and migration of T-ALL cells [23].

Therefore, abnormal microenvironment is the key contributor in the development of leukemia. In this process, various factors, headed by chemokines, play important roles in the progression of ALL via responding to abnormal expression of genes and affecting microenvironments [24]. We further summarize the mechanisms of chemokines and their receptors in ALL.

## The roles of chemokines and their receptors in ALL

**Fig. 1:**
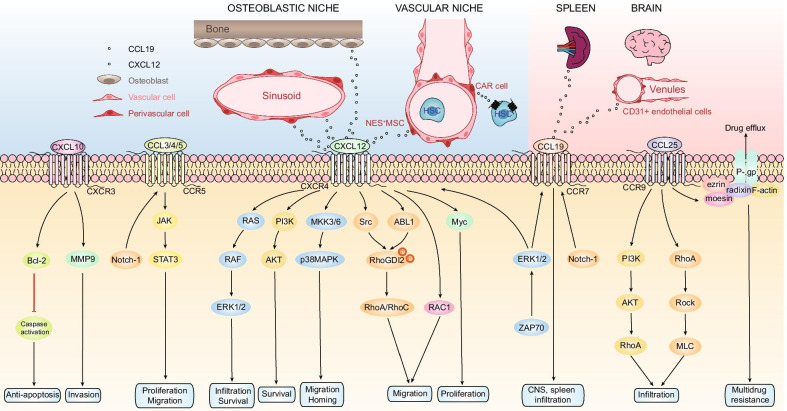
Chemokines and their receptors’ roles in acute lymphoblastic leukemia. In osteoblastic niche, T-ALL cells are in direct, stable contact with vascular cells in the BM that produce CXCL12, while in vascular niche CXCL12 is secreted by several stromal cell types, especially CAR cells. Apart from the BM microenvironment, extramedullary organs such as the brain and spleen showed high expression of CCL19, which is involved in leukemic cells’ infiltration to such sites. CXCL12/CXCR4 triggers the activation of downstream kinases Src and ABL1 which are responsible for the phosphorylation of RhoGDI2, which released RhoA and RhoC, leading to subsequent cytoskeleton redistribution and assembly in the process of migration. CXCR4 could activate both ERK1/2 and PI3K/Akt pathways to promote the survival of leukemic blasts. In the homing of B cell progenitor ALL cells to the BM, CXCL12-mediated activation of p38MAPK was required and ZAP70 kinase can control the expression of CXCR4 and CCR7 via ERK1/2, which is correlated with CNS infiltration during T-ALL. CXCR4 could also activate RAC1 to mediate migration and engraftment of B-ALL cells in the BM or testicles. In addition, CXCR4 can induce the proliferation of T-ALL cells via Myc. CXCL10/CXCR3 axis may increase survival rate of leukemic cells during treatment through stabilizing Bcl-2 and inhibiting caspase activation. CXCL10 has also been proved to promote migration of leukemic cells via MMP9. The expression of CCR5 and CCR7 is regulated by Notch-1. CCR5 regulate leukemic cell’s proliferation and anti-apoptosis through JAK/STAT3 pathway. CCL25/CCR9 axis increase drug efflux-induced resistance by activating the binding of P-gp and ERM. It also facilitates the infiltration through RhoA-Rock-MLC and PI3K/AKT-RhoA pathway

Chemokines, named because of their ability to induce directional chemotaxis in nearby reactive cells, are small cytokines or signaling proteins secreted by cells [25]. They are categorized into four main subfamilies: CXC, CC, CX3C and XC. All of these proteins exert their biological effects by interacting with G protein-bound transmembrane receptors (chemokines receptors), which are selectively expressed on the surface of target cells [26]. Consequently, chemokines play a central role in the development and homeostasis of the immune system [27, 28]. In ALL, different chemokines interacting with their receptors have great effects on the leukemogenesis, progression and relapse. Such kind of relationships between chemokines and the biological process of ALL are exhibited in Fig. 1.

### CXCL12/CXCR4

Stromal cell-derived factor-1 (SDF-1)/CXCL12 functions as the chemoattractant for both committed and primitive hematopoietic progenitors and regulates embryonic development including organ homeostasis [29]. CXCR4 is the cognate receptor of CXCL12, widely expressed in numerous tissues including immature osteoblasts and endothelial cells in the BM, epithelial cells of many organs, CNS and hematopoietic cells [30]. CXCL12/CXCR4 axis provides the stimulus for proliferation, survival, self-renewal, differentiation and functional activation in normal hematopoietic cells [31] and plays the role of retaining normal developing B and myeloid cells in the BM [32].

However, the activation of CXCL12/CXCR4 axis has been found to be involved in various aspects of ALL progression, which includes proliferation, migration, infiltration and chemoresistance. CXCR4 is highly expressed on the surface of mouse and human ALL cells [16, 33–35], and its high expression predicts poor prognosis of ALL patients [36–38]. CXCR4 deletion or treatment with the CXCR4 antagonist could significantly reduce T-ALL burden, indicating that it may be a promising target for ALL therapy [16, 33, 39].

Lots of research has shown that CXCL12/CXCR4 was able to accelerate the proliferation of ALL cells, but the specific mechanism still needs to be further studied [40–44]. Juarez J et al. found that CXCL12 synergized with interleukin (IL) -7 or IL-3 was associated with enhanced phosphorylation of the mitogen-activated protein kinases (MAPK), extracellular signal-related kinase 1/2 (ERK1/2) and p38, and AKT, implicating these pathways in the proliferation of B-ALL [45]. A study based on ALL patients suggested that CXCL12 might enhance proliferation of precursor B-ALL via the signal transducer and activator of transcription 5 (STAT5) activation [46]. Knockout CXCR4 could reduce the expression of Myc, which was responsible for the proliferation of T-ALL cells [16].

Infiltration and chemoresistance are major causes of ALL relapse and correlate with poor prognosis [4, 5]. In the critical process of infiltration, transendothelial migration of leukemic cells is required to exit the blood stream into targeted organs, which is the prerequisite for infiltration [5]. High expression of CXCR4 by leukemic blasts and activation of the CXCL12/CXCR4 axis are involved in leukemia migration [47, 48]. CXCL12/CXCR4 triggered the activation of downstream kinases Src and ABL1 which were responsible for the phosphorylation of Rho GDP-dissociation inhibitor 2 (RhoGDI2) on Y153, Y24 and Y130. Phosphorylation of RhoGDI2 on Y24 and Y153 released RhoA and RhoC from RhoGDI2, which led to subsequent cytoskeleton redistribution and assembly in the process of migration [49, 50]. Besides, p38MAPK signaling was shown to be involved in CXCR4-mediated migration but not proliferation in ALL [40, 51]. CD9 could modulate CXCR4-mediated migration via RAC1 signaling, which may modulate the chemotactic response to CXCL12 by regulating the internalization of CXCR4 [52, 53].

Accumulating data have demonstrated that high expression of CXCR4 is associated with infiltration into spleen, liver, lymph nodes, CNS and testicles in ALL [21, 38]. In the homing of B cell progenitor ALL cells to the BM, CXCL12-mediated activation of p38MAPK was required [51], and those with high CXCR4 expression had more significant liver or spleen infiltration than those with low CXCR4 expression in pediatric B-ALL patients [46]. Infiltration of the CNS is a deteriorate trait of T-ALL [54] and T-ALL neuropathology was found to result from meningeal infiltration through CXCR4-mediated BM colonization [21]. Zeta-chain-associated protein 70 (ZAP70) kinase controlled the expression of CCR7 and CXCR4 via ERK1/2 in B-ALL and T-ALL, which was correlated with CNS infiltration in T-ALL patients [55]. CXCR4 could also activate RAC1 to mediate migration and engraftment of B-ALL cells in the BM or testicles, which was enhanced by CD9 [52].

CXCL12/CXCR4 could induce the chemoresistance of leukemia cells. LICs are thought to be responsible for resistance to treatment and relapse following chemotherapy, and CXCR4 was found to be essential to the LICs activity of T-ALL [43]. In BM leukemic niches, interactions of stromal cells and extracellular matrix with leukemic blasts can generate antiapoptotic signals that provide a sanctuary for subpopulations of leukemic cells to evade chemotherapy-induced death and allow acquisition of a drug-resistant phenotype [47, 56]. Overexpression of CXCR4 granted leukemic blasts a higher capacity to seed into BM niches, and the inhibition of CXCR4 could mobilize the leukemic cells to overcome the therapeutic resistance [57–59]. BM MSCs can protect leukemic cells from chemotherapy via the interplay between CXCL12 and CXCR4. β_1_-integrin/hERG1/CXCR4 complex was found to participate in the interaction between MSCs and ALL cells, activating both the ERK1/2 and the phosphoinositide 3-kinase (PI3K)/Akt pathways to promote the survival of leukemic blasts [60]. The CXCR4 antagonist could effectively reverse MSC-mediated drug resistance and sensitize leukemic cells from refractory/relapsed ALL patients to chemotherapy drugs [61]. Therefore, blocking the CXCL12/CXCR4 axis may have implications for improving the therapeutic effect of ALL patients.

As for the upstream regulation of CXCR4 expression in leukemic cells, runt-related transcription factor 2 which was upregulated in high-risk T-ALL could directly regulate the transcription of *CXCR4* gene, thereby promoted homing to medullary and extramedullary sites [62]. Besides, Kruppel-like factor 4 which was identified as an important negative regulator in T-ALL could directly bind to the promoter of CXCR4 and suppress its expression [63]. Except for transcription factors, ghrelin as a hormone could induce CXCR4 expression via the SIRT1/AMP-activated protein kinase axis in ALL cell lines [64]. CXCR4 could also be suppressed by miRNA-139 which was lowly expressed, whereas CXCR4 was highly expressed in T-ALL cell lines and patient samples [44]. CXCR4 cell surface expression was regulated by cortactin, an actin-binding protein implicated in the regulation of cytoskeleton dynamics, and the expression of cortactin was dependent on calcineurin [43].

### CCL25/CCR9

CCR9 is mainly distributed in immature T lymphocytes and on the surface of intestinal cells, and it plays a role in T lymphocyte development and tissue-specific homing when bound to its specific ligand [65]. CCL25, which is the only ligand for CCR9, is mainly expressed by epithelial cells in the thymus as well as small intestine and acts as an important chemoattractant for T cells in the gut [65–67].

To our knowledge, we are the first to report that CCR9 is highly expressed on T-ALL CD4^+^ T cells, and rarely expressed on normal CD4^+^ T cells [68]. Later studies have found that CCL25/CCR9 axis plays an important role in several aspects of T-ALL progression.

CCR9 is closely related to the infiltration of leukemia cells. Our studies have shown that CCL25 induces MOLT4 cells (human T-ALL cell line with naturally high expression of CCR9) polarization and microvilli absorption to participate in leukemia infiltration and trafficking via the RhoA-Rock-MLC and ezrin pathway [69, 70]. CCL25/CCR9 has also been shown to upregulate the expression of Wnt5a by promoting the expression and activation of protein kinase C, thereby enhancing MOLT4 cells migration, invasion, actin polarization, and lamellipodium and filopodia formation via PI3K/Akt-RhoA pathway activation [71]. We also found that the combined use of IL-2 and IL-4 promoted the internalization of CCR9 and therefore attenuated leukemia cell infiltration and metastasis [72]. Furthermore, Miething C et al. reported that leukemia infiltration into the intestine was dependent on CCR9, which was amplified by PTEN loss, since CCL25 stimulation had little impact on PI3K signaling in the presence of PTEN [73].

CCL25/CCR9 could also induce the chemoresistance of T-ALL. We found that CCL25/CCR9 involvement in the resistance of TNF-α-induced apoptosis in T-ALL depended on Livin, suggesting that CCL25/CCR9 plays an antiapoptotic role [74]. Furthermore, we obtained a multi-resistant T-ALL cell line which was derived from MOLT4 through doxorubicin dosing screening. Then, we investigated this multi-resistant cell line and found that CCR9 induced resistance to chemotherapy drugs, which could be blocked by CCR9 antibodies. Mechanistically, CCL25/CCR9 activated the binding of P-glycoprotein (P-gp) and the cytoskeleton protein ERM to increase P-gp efflux, thus mediating multidrug resistance of T-ALL cells [75].

As for the regulatory mechanism of CCR9 overexpression in T-ALL, it is reported that Notch1 pathway activation could boost the expression of CCR9 [76]. Moreover, we found that certain non-coding RNAs, such as miRNA and lncRNA, may also mediate the expression of CCR9 and further affect its biological function in T-ALL (the relevant work is ongoing).

Therefore, inhibiting CCL25/CCR9 may be a potential therapeutic strategy for treating leukemia patients, and it is of great significance to further explore the role of CCL25/CCR9 in leukemia.

### CXCL10/CXCR3

CXCR3 is preferentially expressed on the surface of monocytes, T cells, NK cells, dendritic cells and cancer cells. CXCL9, CXCL10 and CXCL11 are selective ligands for CXCR3 [77], but so far only the role of the CXCL10/CXCR3 axis has been noted in ALL.

ALL relapse is associated with the survival of blasts in organs such as the CNS or the testicles, where levels of antileukemic drugs are diminished [78]. CXCR3 is highly expressed in cerebrospinal fluid (CSF) leukocytes, and its ligand CXCL10 is upregulated in the CSF of multiple sclerosis patients [79], suggesting that CXCR3 may play an important role in the chemotaxis of cells to CNS.

In T-ALL, the levels of CXCL10 in CSF were found to be significantly higher among patients with CNS relapses. Treating the leukemic mice model with CXCR3 antagonist AMG487 could significantly reduce leukemic infiltration of the CNS [80]. Besides, Williams MT et al. also found that IL-15 might upregulate CXCR3 in precursor B-ALL, and leukemic cells could migrate toward the CXCL10 which is detectable in CSF samples from patients with ALL [81]. These results indicate that CXCL10/CXCR3 axis may promote the CNS infiltration of ALL cells.

In addition, in precursor B-ALL, CXCL10 released by monocytes promoted migration and invasive capacity of CXCR3^+^ precursor B-ALL cells and possibly led to metastatic spread. CXCL10 could induce MMP9 expression and activity in precursor B-ALL cells, which may explain its role in promoting their invasion [82].

CXCL10/CXCR3 axis may also lead to recurrence of ALL by increasing the survival rate of ALL cells during treatment. Mechanistically, CXCL10 enhanced the viability and drug resistance of leukemia cells by stabilizing Bcl-2 and then inhibiting the activation of the caspase cascade induced by drugs such as cytarabine [80]. Therefore, blocking the CXCL10/CXCR3 axis may have a positive effect on reducing relapses in ALL patients.

### CCL19/CCR7

CCR7 and its two ligands, CCL19 and CCL21, play important but apparently opposing roles in cancer. On the one hand, expressed on dendritic cells, T helper cells, Treg cells, B cells and central memory T cells, CCR7 is involved in the modulation of the immune response to a growing tumor. On the other hand, CCR7 also plays a key role in promoting metastasis via the lymphatic system, and it is involved in leukemic cells’ migration to the CNS [83].

Studies have demonstrated that CCR7 could be upregulated by Notch1 and ZAP70/ERK pathway and plays a key role in ALL CNS infiltration [20, 55, 84]. It is worth noting that CCL19 but not CCL21 could be detected in the brain sections of leukemic mice. More specifically, CCL19 is mainly produced by CD31^+^ endothelial cells of brain venules, thereby mediating CNS infiltration in T-ALL [20].

Besides, CCL19 was also highly expressed in the splenic microenvironment, thereby recruiting T-ALL cells with high expression of CCR7. This kind of microenvironment could promote the proliferation of T-ALL cells, stimulate the expression of CCR7 and enhance the migration ability of T-ALL cells to increase the degree of T-ALL malignancy [23].

Moreover, a clinical research indicated that CCR7 was highly expressed in adult acute leukemia cells, and the expression of CCR7 was related to extramedullary infiltration in ALL [85].

### Others

In addition to CXCR4 mentioned above, CXCR7 can also bind to CXCL12, and there is increasing evidence that CXCR7 can activate a series of intracellular signaling pathways by forming a heterodimer with CXCR4 [86, 87], or by clearing and controlling the gradient of CXCL12 to adjust the signal transduction of CXCL12/CXCR4 [88]. CXCR7 was found to be highly expressed in ALL cells, and CXCR7 silencing inhibited migration of T-ALL cells, but did not affect proliferation and apoptosis [89]. However, another study thought that miR-101 targeted CXCR7/STAT3 axis to reduce T-ALL growth and metastasis [90].

The expression levels of CCL2 and IL-8 (CXCL8) were found to be increased in ALL BM microenvironments. These chemokines have adverse effects on normal HSCs by promoting the survival, proliferation and adhesion of BM MSCs, establishing a malignant BM microenvironment, and then resulting in a poor prognosis [91]. Further studies indicated that BM MSCs-derived periostin promoted B-ALL cell proliferation via NF-κB/CCL2 pathway and that B-ALL cells-derived CCL2 increased periostin level in BM MSCs via STAT3 activation [92]. These studies implied critical crosstalk between leukemia cells and stromal cells via CCL2 in ALL progression. Except for the BM microenvironment, clinical samples analysis also indicated that CNS involvement in ALL was associated with significantly higher levels of CCL2 during therapy [93].

CCR5 was also found to be upregulated by Notch1 pathway in ALL cells [76], and the CCR5 selective inhibitor maraviroc can inhibit the proliferation and migration of ALL cells and induce apoptosis by inhibiting the Janus kinase (JAK)/STAT3 pathway [94].

The expression of CCL18, CCL23 could also be seen increased in ALL plasma, which possibly reflects tumor/host cell interactions in the circulation [95]. Gutiérrez-Aguirre CH et al. found that the higher serum XCL1 levels at diagnosis and their progressive decline throughout chemotherapy might be correlated with higher survival, but its mechanism was still unknown [96].

In summary, chemokines and their receptors play an extremely important role in the progression and relapse of ALL. Therefore, exploring the specific mechanisms of chemokines, chemokine receptors and microenvironment, is beneficial to find more drug targets, which can provide a new and effective way for the treatment of ALL patients (Table 1).

**Table 1:** Roles and pathways of chemokines and their receptors in ALL

	Pathways	Roles	References
CXCL12/CXCR4	Myc	Proliferation	[16]
	Src and ABL1/RhoGDI2/RhoA and RhoC	Migration	[49, 50]
	P38MAPK	Migration, homing	[40, 51]
	RAC1	Migration	[52, 53]
	ERK1/2	Infiltration, survival	[55]
	PI3K/Akt	Survival	[60]
CCL25/CCR9	RhoA-Rock-MLC and ezrin	Infiltration	[69, 70]
	PI3K/Akt-RhoA	Infiltration	[71]
	P-gp/ERM/F-actin	Drug resistance	[75]
CXCL10/CXCR3	Bcl-2/caspases	Survival	[80]
	MMP9	Invasion	[82]
CCR5	JAK/STAT3	Proliferation, migration	[94]

## Novel ALL treatment targeting chemokines and their receptors

The first-line treatment for ALL typically includes four phases over 2–3 years: induction, consolidation, intensification and long-term maintenance. Routine CNS prophylaxis is recommended in conjunction with systemic chemotherapy and allogeneic hemopoietic cell transplantation remains the standard consolidation treatment in patients at high risk who are fit and have an available donor [2].

**Fig. 2:**
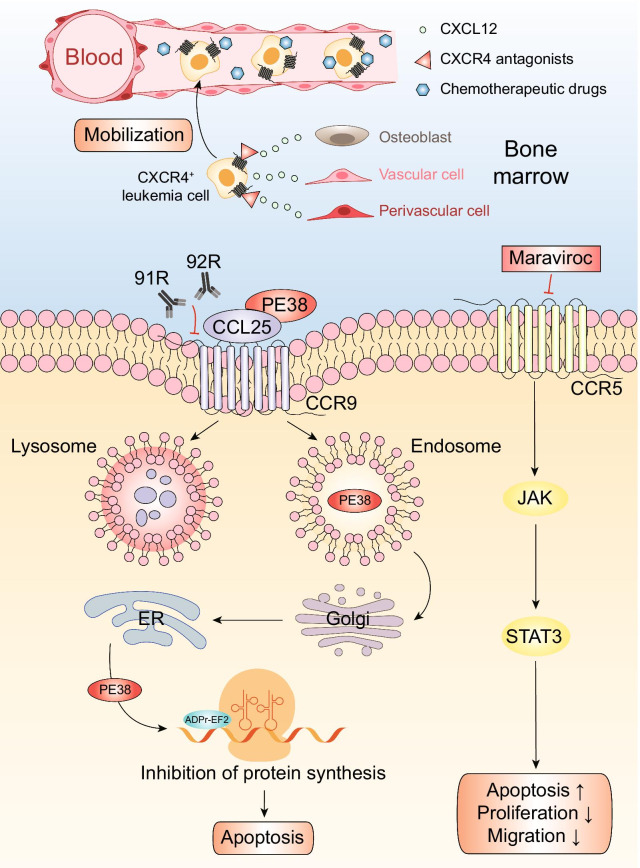
Acute lymphoblastic leukemia’s potential treatments targeting chemokines and their receptors. Chemokine antagonists and immunotoxins combined with chemotherapy may significantly optimize prognosis of ALL patients. CXCR4 antagonists have been proved to inhibit CXCL12-mediated chemotaxis and reverse drug resistance. AMD3100, TC14012 and BL-8040 can block the chemotactic function of the CXCL12/CXCR4 axis, interfere with the bone marrow microenvironment on which leukemia cells depend to survive, and mobilize these leukemia cells into the peripheral circulation, thereby increasing the sensitivity of leukemia cells to chemotherapeutic drugs. CCL25-PE38 fusion protein could effectively induce the apoptosis of CCR9 positive T-ALL cells. Immunotoxin PE38 is internalized via the endolysosomal system, first transported to the Golgi and further to the ER where it is activated. Activated PE38 ribosylates ADP and inactivate EF2, thus halting protein synthesis and eventually leads to apoptosis. Monoclonal antibodies 91R and 92R could inhibit the growth of T-ALL cells transplanted into immunodeficient mice. Competitive antagonist of CCR5 maraviroc inhibits CCR5-activated signaling proteins JAK and STAT3, which may lead to apoptosis, and inhibition of survival, proliferation and migration

Although these therapies have considerably improved outcomes in patients, the main crux in current treatments is the drug resistance of leukemia cells and the high recurrence rate, especially for refractory/relapsed T-ALL, whose therapeutic options are much more restricted [97, 98]. The inefficiency in treatments is closely related to the microenvironment which provides leukemia cells with an ideal environment for survival and proliferation, and thus enables the leukemia cells to escape chemotherapy-induced death [99]. Besides, in the migration and homing of leukemia cells, chemokines and their receptors also play important roles [100]. Based on this view, many inhibitors/biologics targeting chemokine axes, as we have shown in Fig. 2, have shown promising efficacy in preclinical models, and some of them have entered clinical trials.

### Inhibitors of the CXCL12/CXCR4 axis

As the most studied chemokine axis, drugs targeting the CXCL12/CXCR4 axis are the most common and diverse. Some CXCR4 inhibitors are undergoing phased clinical trials. The mechanisms of inhibitors targeting CXCR4 can be summarized as (1) disrupting interactions between adhesion matrices that provide survival signals and drug tolerance signals for leukemia cells; (2) mobilizing tumor cells and making them better accessible to conventional therapy; (3) preventing tumor migration and dissemination; and (4) blocking a series of activation signaling caused by CXCL12/CXCR4 axis activation [32]. Here, we will introduce the drugs separately:

#### AMD3100 (Plerixafor)

AMD3100 is a highly specific antagonist of CXCR4 and blocks binding of CXCL12 by interacting with the carboxylic acid group of Asp^171^ and Asp^262^ at each end of the main ligand-binding crevice [101]. It was shown that AMD3100 specifically inhibited signal transduction initiated by the interaction of CXCL12 with CXCR4 (as monitored by calcium flux) and inhibited CXCL12-induced endocytosis of CXCR4 [101, 102].

In leukemia, AMD3100 can block the chemotactic function of the CXCL12/CXCR4 axis, interfere with the microenvironment on which leukemia cells depend to survive, and mobilize these leukemia cells into the peripheral circulation, thereby increasing the sensitivity of leukemia cells to chemotherapeutic drugs and promoting the apoptosis of leukemia cells [103]. AMD3100 could also effectively reverse MSCs-mediated drug resistance and sensitize leukemic cells from refractory/relapsed ALL patients to chemotherapy drugs [61]. Moreover, AMD3100 increased the proportion of activated parts of the cell cycle, which may increase the sensitivity of cell cycle-dependent drugs in the treatment of ALL, such as vincristine [104].

AMD3100 inhibited the proliferation of extramedullary ALL cells after chemotherapy and greatly improved the OS rate of AMD3100-treated mice, and reduced the recurrence rate of ALL [22]. It was also found to inhibit CXCL12 induced migration and phosphorylation of ERK 1/2 in leukemia mouse models [105].

In a phase 1 study (NCT01319864), pediatric patients with refractory/relapsed acute myeloid leukemia (AML), ALL or myelodysplastic syndrome (MDS) were administered AMD3100 and combination chemotherapy consisting of etoposide and cytarabine daily for five days. However, there were only moderate clinical responses in three patients with AML and no responses were observed in patients with ALL or MDS [106]. Main defects of AMD3100 include a short half-life and limited method of administration, which is only injection-administered [107].

#### AMD11070

AMD11070 is a non-cyclamin orally administrable CXCR4 antagonist that can inhibit CXCL12-induced signaling pathways in ALL cells and block the migration of ALL cells towards CXCL12 and stroma. Combination treatment of AMD11070 with chemotherapeutic drugs reduces recovery from treatment [108]. In vitro experiments confirmed that the same concentration of AMD11070 showed stronger efficiency than AMD3100 in the recognition of CXCR4 and the chemotaxis effects of ALL cells to CXCL12 [108]. A comparative study of AMD3100 and AMD11070 proved that these two compounds bond to overlapping but not identical residues in the receptor-binding regions [109]. Therefore, AMD11070 and AMD3100 have different receptor affinities, binding constants and abilities to block multiple downstream effects in which the receptor CXCR4 participates.

A phase I study confirmed that the use of AMD11070 did not cause safety problems [110], but pathological damage to the liver had been observed in long-term animal experiments [111]. Therefore, the optimal clinical dose of AMD11070 still needs further determinations.

#### AMD3465

AMD3465 antagonizes the binding of CXCL12 and inhibits the CXCL12-mediated signal transmission process including GTP binding, calcium flux and chemotaxis. At the same time, it has been shown to cause leukocytosis in animal experiments of mice and dogs, suggesting that it may possess the potential for HSC mobilization [112].

Pitt LA et al. confirmed that AMD3465 alone could effectively reduce the number of ALL cells in the spleen, thymus, BM and lymph nodes in ALL mice, thus implicating the antileukemia effect of this CXCR4 antagonist even when administered alone, providing a potential treatment strategy for ALL [16].

#### T140, T134 and TC14012

The polyphemusin II analogs T140, T134 and TC14012 are potent inhibitors of CXCL12-mediated chemotaxis and the BM-dependent B-ALL progenitor cells proliferation [113]. T140 attenuated the migration of pre-B-ALL cells into BM stromal layers in vitro, while TC14012 significantly inhibited stroma-dependent proliferation and enhanced the cytotoxic and antiproliferative effects of the cytotoxic agents vincristine and dexamethasone [113]. In the mouse model, CXCR4 antagonists AMD3100 and TC14012 can interfere with B-ALL progenitor cells and their supporting niches in vivo by blocking the interaction of CXCL12/CXCR4 and mobilize the leukemia cells rapidly into the peripheral blood therefore reducing their numbers at the local lesion [114].

#### BL-8040

The T140 analog BL-8040 is a synthetic peptide antagonist with a high affinity and a slow dissociation rate from CXCR4 [115]. It has been reported that BL-8040 potently suppresses T-ALL cell growth in xenotransplantation models of T-ALL. In an ongoing phase II trial (NCT02763384) involving patients with refractory/relapsed T-ALL, BL-8040 is being administered in combination with nelarabine. Five of nine patients enrolled in the study have achieved a complete remission with an overall response rate of 56%. The study also showed the sustained blockade of CXCR4 on leukemic blasts at 24 h after administration along with mobilization of ALL blasts into the peripheral blood [116].

#### POL5551

The protein epitope mimetic POL5551 is a novel CXCR4 antagonist [117]. This medium-sized, fully synthetic cyclic peptide-like molecule functions via mimicking the two most critical motifs in protein interactions, β-hairpins and α-helices [118]. Sison EA et al. first reported POL5551 in the study of pediatric ALL, showing that POL5551 was an inhibitor more potent than plerixafor. POL5551 exerted its effects through binding to CXCR4 surface at the 12G5- (and thus CXCL12-) binding site, resulting in the attenuation of CXCL12-mediated phosphorylation of ERK1/2, inhibition of CXCL12 induced chemotaxis, and restoration of chemosensitivity in a stroma co-culture model [119]. Thus, POL5551 may improve the outcomes in high-risk refractory pediatric ALL.

### Inhibitors of the CCL25/CCR9 axis

#### CCL25-Pseudomonas exotoxin (PE38) fusion protein

We previously developed a CCL25-PE38 fusion protein using genetic engineering, which was proved to be able to attract the migration of MOLT4 cells and then induce the apoptosis of these cells thus suppressing the growth of CCR9^+^ tumors [120].

The induction of apoptosis is achieved through the internalization of PE38 when bound via the endolysosomal system, by which it is first transported to the Golgi and further to the endoplasmic reticulum and get activated. The activated PE38 ribosylates ADP and inactivates elongation factor 2 (EF2), thus halting protein synthesis and eventually leads to apoptosis [65, 121].

CCL25-PE38 could induce the specific killing of CCR9^+^ cells, but shown no cytotoxicity on CCR9^−^ cells [120]. Optimization of the structure of CCL25-PE38 as well as increasing its activity and stability may make CCL25-PE38 a superior reagent in the treatment of T-ALL.

#### Monoclonal antibodies 91R, 92R

Monoclonal antibodies have been a standard component of cancer therapy, which work through several mechanisms, including antibody-dependent cell-mediated cytotoxicity (ADCC), complement-dependent cytotoxicity (CDC) and direct induction of apoptosis [122, 123].

Monoclonal antibodies 91R (mouse antihuman CCR9 IgG2b) [124] and 92R (mouse antihuman CCR9 IgG2a) [125] were shown to inhibits the growth of T-ALL cells transplanted into immunodeficient mice, and both of them were able to elicit CDC and ADCC in vitro against CCR9^+^ cells. These antibodies have therapeutic potential for the targeted elimination of CCR9^+^ tumor cells, used either alone or in combination with other therapies.

### Inhibitor of CCR5

CCR5 inhibitor maraviroc has been widely used in the treatment of HIV [126, 127], and recent studies have shown that it has potent inhibitory effects on many different tumors [128]. Treatment with maraviroc could inhibit the survival, proliferation and migration of ALL cells in vitro and in vivo, which may be achieved by inhibition of JAK/STAT3 pathway, a major signaling pathway that plays a role in the development of leukemia [94]. Therefore, the CCR5 inhibitor maraviroc may be a potential human ALL therapeutic agent.

### Others

CXCR7 has showed great relevance to CXCR4, which indicates the inhibitor of CXCR7 may accomplish surprising outcomes. Ando N et al. found that application of CXCR7 inhibitor can accelerate the apoptosis of ALL cells with MLL gene rearrangements [88].

Besides, Tan Q et al. developed diphtheria-toxin-based antihuman CCR4 immunotoxins, which significantly prolonged the survival of tumor-bearing mice injected with human CCR4^+^ ALL cells [129].

Therefore, combining chemokine antagonists to the conventional therapy may significantly optimize prognosis of ALL patients [56]. Table 2 summarizes the potential chemokine antagonists for the treatment of ALL.

**Table 2:** Potential therapies targeting chemokines and their receptors in ALL

Targets	Drugs	Clinical stages/NCT numbers	Functions	References
CXCL12/CXCR4 axis	AMD3100 (Plerixafor)	Phase 1/ NCT01319864	Increasing the sensitivity of chemotherapeutic drugs and promoting the apoptosis of ALL cells	[103]
	AMD11070	NA	Inhibiting CXCL12-induced signaling pathways and block the migration of ALL cells	[108]
	AMD3465	NA	Suppressing the growth and infiltration of T-ALL	[16]
	T140, T134 &TC14012	NA	Inhibiting proliferation and migration, enhancing the effects of the cytotoxic agents	[113, 114]
	BL-8040	Phase 2/ NCT02763384	Suppressing T-ALL cell growth in xenotransplantation models and mobilizing of leukemic blasts into the peripheral blood in T-ALL patients	[116]
	POL5551	NA	Attenuating CXCL12-mediated phosphorylation of ERK1/2, inhibiting CXCL12-induced chemotaxis, and increasing chemosensitivity of ALL cells	[119]
CCL25/CCR9 axis	CCL25-PE38	NA	Inducing apoptosis and inhibiting the growth of CCR9^+^ cells	[120]
	91R, 92R	NA	Inhibiting CCR9^+^ T-ALL tumor growth through CDC, ADCC	[124, 125]
CCR5	Maraviroc	NA	Inhibiting the proliferation of ALL cells by inhibiting the JAK/STAT3 pathway	[94]

NA: Not applicable

## Conclusions

ALL is the most common childhood malignancy, accounting for 20% of all cancers before 20 years of age [130]. With the risk stratification based on biological features of ALL cells and response to treatment, the treatment modification adapted from pharmacodynamics and pharmacogenomics, and the improved supportive care, 5-year OS in pediatric ALL has improved to roughly 90% in clinical trials [131]. However, relapse and refractory of ALL remain obstacles, making it necessary to look for the clarification of the mechanism of ALL occurrence and relapse.

Infiltration and chemoresistance of ALL cells are main causes of the relapse, which at least are partly dependent on signals between the interactions of leukemic microenvironment and chemokines. In general, with the existing of chemokines and their receptors, leukemia cells tend to have the traits of migration and infiltration, and at the same time, the leukemic microenvironment can provide shelter where leukemic cells escape chemotherapy-induced death and acquire a drug-resistant phenotype. In addition, the refractory nature of ALL is also related to the protective effect of the microenvironment, where chemokines rebuild the healthy one into a “leukemic” one, thereby destroying normal hematopoietic function. Therefore, using chemokines and their receptors as targets for new drugs will become an effective method for the improvement of therapeutic efficacy and patient prognosis.

However, the physiological functions of chemokines and their receptors cannot be ignored, which may be correlated with the side effects of targeting chemokines. For example, CXCL12/CXCR4 is also important to HSCs maintenance within BM, and antagonism of CXCL12/CXCR4 could lead to HSCs mobilization out of BM into the peripheral blood, so the safety of the drugs should also be carefully evaluated [132].

Although many studies have shown the important roles of chemokines and their receptors in ALL, the exact mechanisms remain to be further elucidated and more clinical trials are needed to confirm their effects. In general, with the deeper understanding of chemokines roles in ALL, we believe that more and more ALL patients will benefit from the development of novel targeted therapies in the near future.

## Data Availability

Not applicable.

## Abbreviations

ALL: Acute lymphoblastic leukemia
OS: Overall survival
EFS: Event-free survival
BM: Bone marrow
HSCs: Hematopoietic stem cells
CNS: Central nervous system
MSCs: Mesenchymal stem cells
Treg cells: Regulatory T cells
SDF-1: Stromal cell-derived factor-1
IL: Interleukin
MAPK: Mitogen-activated protein kinases
ERK1/2: Extracellular signal-related kinase 1/2
STAT: Signal transducer and activator of transcription
RhoGDI2: Rho GDP-dissociation inhibitor 2
LICs: Leukemia-initiating cells
PI3K: Phosphoinositide 3-kinase
P-gp: P-glycoprotein
CSF: Cerebrospinal fluid
JAK: Janus kinase
AML: Acute myeloid leukemia
MDS: Myelodysplastic syndrome
PE38: Pseudomonas exotoxin
EF2: Inactivates elongation factor 2
ADCC: Antibody-dependent cell-mediated cytotoxicity
CDC: Complement-dependent cytotoxicity
